# Changes in the accessibility of the HIV-1 Integrase C-terminus in the presence of cellular proteins

**DOI:** 10.1186/1742-4690-7-27

**Published:** 2010-04-05

**Authors:** Sofia Benkhelifa-Ziyyat, Stéphanie Bucher, Maria-Antonietta Zanta-Boussif, Julie Pasquet, Olivier Danos

**Affiliations:** 1 Généthon, 1 rue de l'Internationale, Evry, 91002, France; 2 Inserm U951, Université d'Evry Val d'Essonne, Généthon, 1 rue de l'Internationale, Evry, 91002, France; 3 Inserm U781, Université Paris Descartes Hôpital Necker-Enfants Malades, 149 rue de Sèvres, Paris, 75015, France

## Abstract

**Background:**

Following entry, uncoating, and reverse transcription, a number of cellular proteins become associated with the Human Immunodeficiency Virus type 1 (HIV-1) pre-integration complex (PIC). With the goal of obtaining reagents for the analysis of the HIV-1 PIC composition and localisation, we have constructed functional integrase (IN) and matrix (MA) proteins that can be biotinylated during virus production and captured using streptavidin-coated beads.

**Results:**

Although the labelled C-terminus allows for the sensitive detection of virion-associated IN, it becomes inaccessible in the presence of cellular proteins. This masking is not dependent on the nature of the tag and does not occur with the tagged MA. It was not observed either with an IN mutant unable to interact with LEDGF/p75, or when LEDGF/p75 was depleted from cells.

**Conclusion:**

Our observation suggests that a structural rearrangement or oligomerization of the IN protein occurs during the early steps of infection and that this process is related to the presence of LEDGF/p75.

## Background

Integration of the Human Immunodeficiency Virus (HIV) DNA into the host cell chromosome mediated by the integrase (IN) protein is an obligatory step of the virus life cycle. This endonuclease encoded by the pol gene generates active CA-3'-hydroxyl ends on the viral cDNA and catalyses strand transfer with the chromosomal DNA. IN is also involved in the processing and trafficking of the viral genome throughout the pre-integration phase including reverse transcription and nuclear import [1-3]. The IN protein is organized in three domains: an N-terminal domain (NTD) involved in higher order multimerization (residues 1-49), a catalytic core domain (CCD) (residues 50-212) and a C-terminal domain (CTD) (residues 213-288) with DNA binding activity. IN activity is modulated by its interactions with viral and cellular proteins within the Pre-Integration Complex (PIC) [1,2]; these interactions protect it from degradation [4,5], target it to the relevant cell compartment [6,7] and enhance its catalytic activity [1,8,9]. Among the cellular partners of IN, the most studied and characterized is LEDGF/p75 [1,8,10], a stress-induced transcription co-activator that binds the IN CCD [11,12] and tethers the viral cDNA to transcriptionally active regions of the genome [13]. PICs have not been fully characterized yet due to the limited quantity of material that can be purified from HIV infected cells. Yet, a complete identification of PIC components could provide new targets for antiviral therapy and help to target the integration of lentiviral vectors used in gene therapy [14]. Our initial goal in this study was to generate a tagged integrase that could be biotinylated for streptavidin-mediated capture and purification of PICs. Our data indicate that an active C-terminally tagged IN can be generated and efficiently incorporated into virions. However, we show that the C-terminal tag is not accessible for capture in the context of the PIC. This masking of the IN C-terminus is dependent on the presence of LEDGF. It is consistent with a structural remodelling of IN that is believed to occur during PIC formation in HIV infected cells.

## Results

### Production and characterization of an HIV-based lentiviral vector containing a tagged integrase

**Figure 1:**
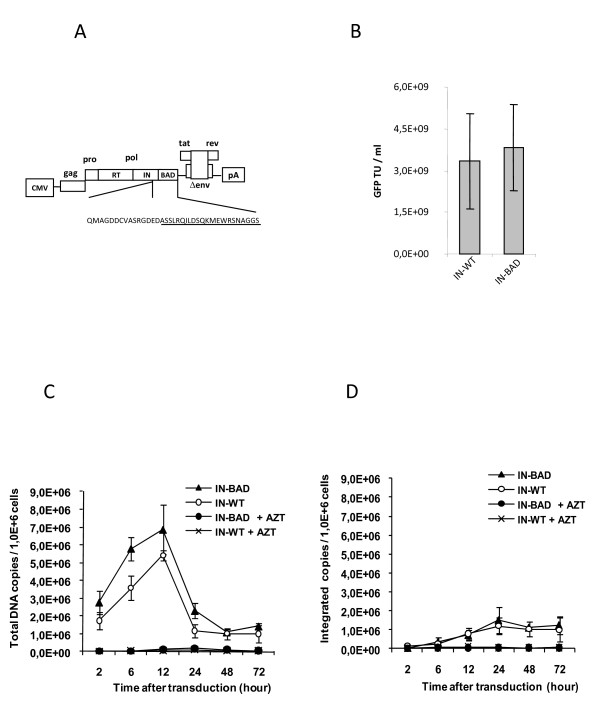
**Fusion of the Biotin Acceptor Domain (BAD) to the IN C-terminus does not affect particle production, cDNA synthesis, and integration**. (A) Amino acid sequence at the C-terminus of IN-BAD, in the context of a p8.74 derived gagpol expression construct. (B) Comparison of vector titres obtained with IN-BAD and IN-WT. Data represent the mean ± SD of GFP titres measured on HCT116 cells from three independent productions. (C) Kinetics of HIV-1 vector DNA synthesis during vector transduction of HEK 293 cells (30 ng of p24^gag^/10^6 ^cells) with or without AZT, analysed by quantitative PCR. (D) Amounts of integrated provirus. Data in C and D represent the mean ± SD of three independent transductions.

We tagged HIV-1 IN at its C-terminus by adding a 22 amino-acid Biotin Acceptor Domain (BAD) which can be biotinylated *in vivo *in the presence of Bir A, a biotin ligase from *E. coli *[15,16]. A VSV-G pseudotyped lentiviral vector encoding GFP was prepared using gag-pol expression constructs with either the wild-type (IN-WT) or the tagged IN (IN-BAD) sequence (Fig. 1A), and a construct expressing the BirA gene was included in all lentiviral vector preparations. The presence of the BAD tag and its biotinylation by BirA did not affect the amounts of p24^gag ^antigen released from transfected cells (not shown) nor the vector titre measured in GFP transducing units (Fig. 1B). The kinetics of viral DNA synthesis (Fig. 1C) and integration (Fig. 1D) determined by PCR [17] over 72 hours following transduction were identical for IN-BAD and IN-WT vectors. We concluded that the activity of the tagged IN was undistinguishable from that of the parental protein.

### Biotinylation and capture of IN-BAD

**Figure 2:**
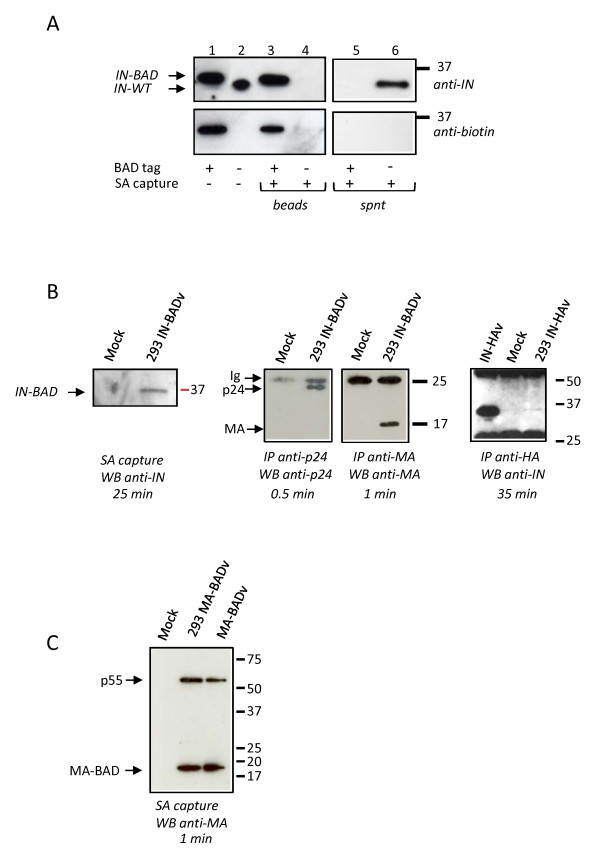
**IN-BAD is efficiently biotinylated in producer cells and incorporated into virions**. IN-BAD (lanes 1, 3) or IN-WT (lanes 2, 4) vector particles (30 ng of p24^gag^) were either untreated (lanes 1, 2) or incubated with streptavidin paramagnetic beads and eluted (SA capture, lanes 3, 4). Samples were run on SDS-PAGE and Western blots (WB) were analysed with anti-IN (top) or anti-biotin (bottom) antibodies (1 minute exposure). Supernatants (spnt) from SA captures were also analysed (lane 5 and 6). (B) Left panel: streptavidin paramagnetic beads capture (SA capture) of the biotinylated IN (IN-BAD) from extracts of 293 cells mock-transduced (Mock) or transduced with the IN-BAD vector (293 IN-BADv), analysed by Western blotting with an anti-IN antibody. Middle panels: as controls, MA or p24 were immunoprecipitated (IP) respectively with an anti-MA and an anti p24 antibodies from the same cells extracts and analysed by WB respectively with the same antibodies. Right panel: HA tagged integrase (IN-HA) was immunoprecipitated with an anti-HA antibody from lysed IN-HA vector (IN-HAv) or from extracts of 293 cells mock-transduced (Mock) or transduced with IN-HAv (293 IN-HAv) and analysed by Western blotting with an anti-IN antibody. (C) Streptavidin paramagnetic beads capture of the biotinylated MA (MA-BAD) from extracts of 293 cells mock-transduced (Mock) or transduced with the MA-BAD vector (293 MA-BADv), or from lysed MA-BAD vector (MA-BADv) analysed by Western blotting with an anti-IN antibody.

IN-BAD and IN-WT vector preparations were analysed by Western blot using anti-IN or anti-Biotin antibodies. Figure 2A shows that the tagged integrase displaying the expected size difference was correctly incorporated into virions and biotinylated (lane 1). Comparable amounts of tagged and wild-type integrase were present in the respective virions, indicating that the BAD addition did not affect viral proteins synthesis and assembly. We tested the possibility to capture the tagged integrase by lysing virions and incubating them with paramagnetic streptavidin-coated beads. Bound material was eluted and analysed by Western blot. The data in Figure 2A (lanes 3 and 4) indicate an efficient and specific capture of IN-BAD on streptavidin beads. IN-BAD was not recovered from the unbound fraction, contrary to IN-WT, indicating a very efficient capture (Fig. 2A, lanes 5 and 6).

### Capture of IN-BAD from lysates of infected cells

HEK 293 cells were transduced with the IN-BAD vector (IN-BADv) or mock-transduced, and whole cell extracts were prepared, as described in Materials and methods, and incubated with streptavidin-coated beads. The eluted material was analysed by Western blot. Figure 2B demonstrates the selective SA capture of the biotinylated IN from cell extracts (left panel). However, this capture was inefficient, with an average of 30 minutes exposure needed to visualize the protein in repeated experiments. No associated LEDGF/p75 could be revealed when the membrane was reprobed with an anti-LEDGF/p75 antibody (not shown). Control immunoprecipitations (IP) indicated that both MA and p24 proteins were readily detected in the same cell extracts (Fig. 2B, middle panels). The experiment was repeated using a lentiviral vector in which the integrase was C-terminally tagged with an HA epitope (IN-HAv) (see Materials and methods). Here again the integrase was efficiently immunoprecipitated with an anti-HA antibody from the lysed IN-HAv, but was poorly pulled down by the same antibody from HEK 293 cells transduced with the IN-HAv (Fig. 2B right panel). Finally, when a BAD tag was inserted into the MA protein (see Materials and methods), the MA-BAD was incorporated into virions (MA-BADv) and efficiently recovered from infected cells using the same conditions of transduction, lysis, and SA capture used in the IN-BAD experiment (Fig. 2C). As a control, we checked that when IN-BAD virions were applied to HEK 293 cells at 4°C for 4 hours before washing with K buffer, no viral material was detected in the cell lysate in pull down experiments (not shown). We concluded that the biotinylated tag at the C-terminus of the IN protein, which can be detected in virions, becomes inaccessible for streptavidin binding after entry into the cell.

### Efficient co-immunoprecipitation of integrase and LEDGF/p75

**Figure 3:**
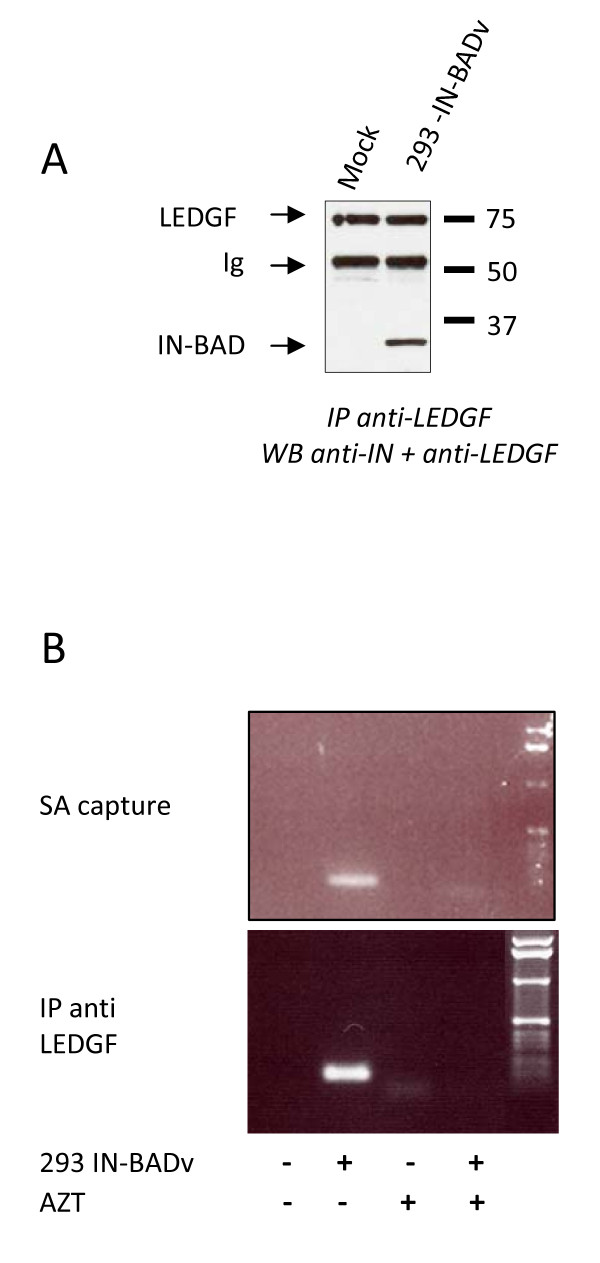
**(A) IN-BAD and LEDGF/p75 co-immunoprecipitation from extracts of 293 cells mock-transduced (Mock) or transduced with the IN-BADv (293 IN-BADv), analysed by Western blotting with anti-LEDGF/p75 and anti-IN antibodies (1 minute exposure)**. (B) PCR detection of viral DNA in streptavidin capture (top) and LEDGF/p75 immunoprecipitates (bottom). 293 cells were transduced with the IN-BAD vector (293 IN-BADv) or mock transduced (Mock) in the absence or presence of AZT. Streptavidine capture or LEDGF/p75 co-immunoprecipitation were performed on cell lysates, and vector DNA was detected using PCR with the MH531 and MH532 primers [17]. The absence of amplification in the presence of AZT indicates that only neo-synthesized DNA was detected.

The minute amount of pulled-down IN could have been due to an early dissociation from PICs and degradation or due to masking of the biotinylated tag in the context of PICs. To resolve these issues, we analysed the presence of IN in our samples (the same extract used in SA capture experiment shown in Fig. 2B) by co-immunoprecipitation with LEDGF/p75, which is reportedly associated with functional PICs [18]. Using this approach, the IN-BAD was readily detected (1 minute exposure) in HEK 293 IN-BADv (Fig. 3A). This indicated that IN had not been degraded, but rather was kept in a configuration where the biotinylated tag could not react with streptavidin. PCR analysis on the pulled down material from the anti LEDGF/p75 IP shown in Fig. 3A or from the SA capture shown in Fig. 2B indicated that the viral DNA was associated with the integrase, whether LEDGF/p75 was present (co-immunoprecipitation) (Fig. 3B, bottom) or not (SA capture) (Fig. 3B, top). Negative PCR controls included transductions made in the presence of azidothymidine (AZT) (Fig. 3B) as well as immunoprecipitation with Protein A beads alone, or a control IgG1 isotype, or a p24 monoclonal antibody which does not precipitate PICs (not shown).

### The presence of LEDGF/p75 in infected cells prevents access to the IN C-terminus

**Figure 4:**
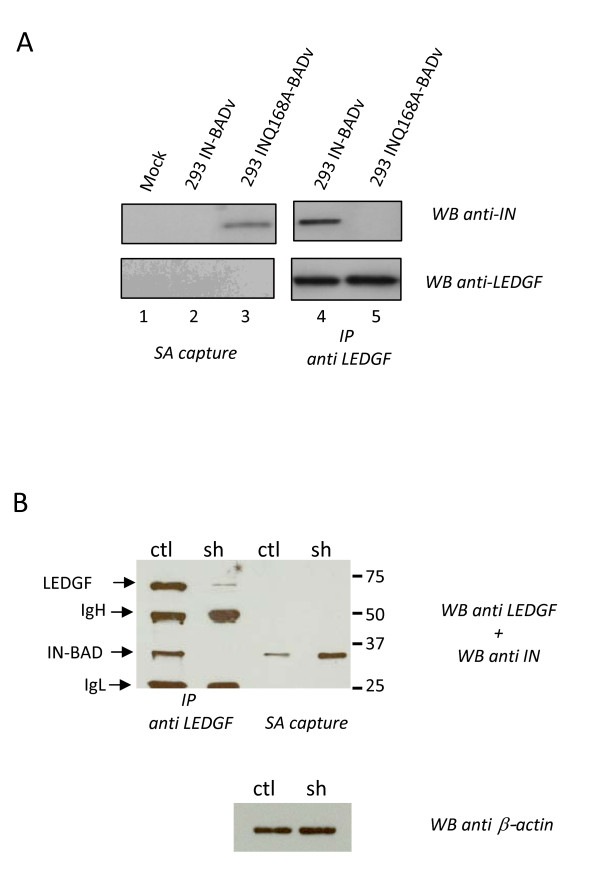
**Masking of the IN C-terminus in infected cells**. (A) Streptavidin paramagnetic beads capture (SA capture) (1,2,3) or LEDGF/p75 co-immunoprecipitation (4,5) of the biotinylated IN from extracts of 293 cells mock-transduced (lane 1) or transduced with the IN-BAD vector (293 IN-BADv) (lane 2, 4) or INQ168A-BAD vector (293 INQ168A-BADv (lane 3, 5) analysed by Western blotting with the anti-IN (top) or anti LEDGF/p75 (bottom) antibodies (3 minutes exposure). (B) LEDGF/p75 co-immunoprecipitation or streptavidin capture of the biotinylated integrase from extracts of 293^ctl ^(ctl) or 293^sh ^(sh) mixed with the IN-BAD vector (IN-BADv). As a control, equal amount of 293ctl or 293sh lysates were tested for beta-actin content by WB (bottom panel).

We next asked whether the presence of LEDGF/p75 in cells lysates could be linked directly or indirectly to the masking of the IN C-terminal tag. Transductions of HEK 293 cells and streptavidin beads capture from cell lysates were repeated with IN-BAD virions containing a Q168A mutant of IN (INQ168A-BADv). This mutation modifies the interface between LEDGF/p75 and the IN binding domain and, depending on the assay, abrogates or severely reduces the interaction with LEDGF/p75 [10,11,19]. The data shown in Figure 4A confirmed the absence of detectable interaction between the INQ168A-BAD and LEDGF/p75 in infected cells (293 INQ168A-BADv, Fig. 4A lane 5). Another clear effect of the IN mutation was to render the IN C-terminus accessible for SA capture (Fig 4A, lane 3).

These data were confirmed using LEDGF/p75 depleted cells lysates. HEK 293 cells were transduced with a lentiviral vector encoding GFP and a LEDGF/p75 shRNA [20] (HEK 293^sh ^cells) or with a control vector (HEK 293^ctl ^cells). GFP^+ ^populations were generated and analysed for vector genome copy numbers by qPCR and LEDGF/p75 protein expression by Western blot. Cell populations with around 10 copies of the vector genome that expressed more than tenfold reduced levels of LEDGF/p75 were subsequently used (sh, Fig. 4B). Reduced levels of LEDGF/p75 were associated with slow growth and increased cell death, as previously described in attached cells [21,22]. Lentiviral transduction of these LEDGF/p75 depleted cells was highly toxic, precluding attempts to capture IN-BAD from lysates of infected cells. Instead, we mixed lysates obtained from IN-BAD particles (IN-BADv) and HEK 293 cells (293^ctl ^or 293^sh^) and asked whether IN-BAD could be captured on streptavidin beads. IN-BAD co-immunoprecipitations with LEDGF/p75 were performed as controls. As expected, IN-BAD could be co-immunoprecipitated with LEDGF/p75 when the IN-BADv was mixed with an HEK 293^ctl ^cells lysate, but not with the HEK 293^sh ^lysate (Fig. 4B). The masking of the IN-BAD C-terminus was again observed when lysed IN-BAD particles were mixed with an HEK 293^ctl ^lysate. In contrast the capture was improved at least 9 fold when an HEK 293^sh ^cell lysate was used. Altogether these results confirm that the IN-BAD C-terminus is masked in the presence of LEDGF/p75 protein in cell lysates

## Discussion

The possibility to tag HIV-1 integrase without affecting infectivity would allow its use as bait to purify and analyse PICs composition by biochemical methods [15,23,24]. Here, we have added a biotinylable tag at the C-terminus of IN (IN-BAD) and showed that the protein remains fully active in the context of a lentiviral vector. The kinetics of viral DNA synthesis and integration were identical for IN-BAD and IN-WT vectors in HEK 293 cells. IN-BAD is efficiently biotinylated and captured from virions on streptavidin coated beads. Unexpectedly it is not efficiently pulled down from infected cells, whereas it remains readily co-immunoprecipitated with LEDGF/p75. The biotin tag-mediated capture is however improved when LEDGF/p75 interaction is abrogated either by a Q168A-IN mutation or by LEDGF/p75 depletion from cells.

The addition of a biotinylable tag to the C-terminus of IN and to MA has recently been reported in the context of an infectious HIV-1_NLX _clone (respectively NLXIN_B _and NLXMA_B_). While tag insertion in MA was well tolerated, the C-terminal tagging of IN resulted in 40% reduction in the virus titer in MAGI-5 cells and in integrase activity *in vitro *[15]. In SupT1 cells, replication kinetics of NLXIN_B _is delayed in comparison to either NLX or NLXMA_B_. Furthermore the biotinylation of the tagged integrase rendered this virus non-infectious in MAGI-5 cells. The difference with our result may be explained by the fact that experiments were conducted with different viral and IN-tag nucleotide and protein sequences. In the context of HIV-1NLX, the insertion of the tag introduced a stop codon in the overlapping vif gene. Although vif activity is irrelevant in the context of SupT1 and MAGI cells, the modification may have cis-acting consequences, for instance on mRNA splicing. More importantly, the sequence of our pol-BAD junction is different from that of Belshan *et al*., who introduced 4 additional amino acids (Leu Gly Gly Ser) at the C-terminus of IN, upstream of the BAD [15]. Such a minor difference may have an important impact, as it is established that C-terminal modifications or tagging of the HIV-1 IN may render the protein sensitive to additional modifications. For example the K(264/266/273)R mutation of IN is without effect on viral replication unless a C-terminal tag is added [25].

C-terminally-tagged IN has been used to probe interactions with cellular proteins upon ectopic expression, leading to the identification of LEDGF/p75 as the major interactor [2,8,18,26]. We show here that LEDGF/p75 readily interacts with a naturally processed IN-BAD present in virions and PICs. We confirm that this interaction is DNA independent, and we observe that it limits the accessibility of the IN C-terminus. The Integrase Binding Domain (IBD) of LEDGF/p75 interacts with the IN-CCD, but no interaction with the IN-CTD has been documented [11,12,27]. It is therefore likely that the masking we observe is indirect and due to a conformational change of IN induced by LEDGF/p75 binding. The three IN domains are connected by flexible linkers which probably allow a conformational variability and different oligomerization states and catalytic properties [28]. For instance, it was shown that IN can undergo a metal dependent conformational change, which results in the loss of recognition by CCD and CTD-specific antibodies [29,30]. Moreover, a DNA-induced protein conformational change leading to connection of these two domains has recently been described [31,32]. The Michel *et al*. study [31] describes an intramolecular contact of the IN-NTD with the IN-CTD in a complex containing 4 IN and 2 LEDGF/p75 molecules, which represent the catalytically active form of the integrase [33,34]. The IN-CTD is also known to contribute to IN multimerisation [35] and promotes binding to different cellular proteins (Gemin2, importin7, APOBEC3G, EED, p300) [26,36-39]. Our data show that integrase capture from cell lysates through a C-terminal tag is significantly improved when LEDGF/p75 is depleted or when IN-LEDGF/75 interaction is abrogated. We suggest that this change in accessibility of the C-terminus reflects a LEDGF/p75 associated structural reorganization of the protein.

In our experiment, LEDGF/p75 was not detected in association with the small amounts of integrase attached to streptavidin beads suggesting that only a LEDGF/p75-free integrase may display an accessible C-terminal tag. C-terminal masking was not detected in studies where IN was over-expressed in cell lines [8,10,18]. Given the high concentration of IN expressed in these cells, the stoichiometry of the interacting partners must be significantly different from physiological conditions in infected cells. The virion and PICs associated IN that we study here are naturally cleaved from the gag-pol precursor and are present at low concentrations. The virion-borne IN may also carry modifications which are not present on the ectopically expressed one. We propose that depending on the experimental system, two types of IN-LEDGF/p75 complexes may form: one in which the C-terminus is accessible requiring high IN concentrations, and possibly IN oligomerization; and another one, mainly represented in infected cells at low and physiological IN concentrations where the C-terminus is masked. Unmasking at high IN concentration could be due to a structural rearrangement led by the titration of a second cellular partner whose concentration is limiting and/or by the absence of other viral components of the PIC like MA and reverse transcriptase (RT). Indeed, the RT protein which was shown to be a PIC component interacts with the IN CTD [40-42].

## Conclusions

The addition of a biotinylable tag to the HIV-1 integrase has allowed us to observe a dynamic change in the protein that takes place during the early steps of viral infection. This change is dependent on an interaction with LEDGF/p75. Understanding its significance awaits further progress in the characterization of the cellular partners of PICs as well as the resolution of the complete PIC structure.

## Methods

### Plasmids

The birA biotin ligase gene (NCBI accession number AF044308) was amplified from *E. coli *genomic DNA by PCR and introduced into the pcDNA (Invitrogen) expression plasmid. For gag-pol expression constructs, a 22 amino-acid biotin acceptor domain (BAD) (Fig. 1A) [16] was introduced in the pCMVΔR8.74 [43] either at the C-terminus of IN (pCMVΔR8.74-IN-BAD) or in the N-terminal region of MA. For pCMVΔR8.74-IN-BAD, a 450 pb IN fragment (F1) was PCR amplified with the following primers (S1: 5' TTTGGCATTCCCTACAATCC3'), and (AS1: 5'**CCAGAATTTGACGCAGAGAAGAAGC***ATCCTCATCCTGTCTACTTGCC *3', including the 22 terminal nt of IN in italics and 25 nt of the BAD sequence, underlined). Oligonucleotides corresponding to the complete BAD sequence plus 10 nt at the 3' end of IN were annealed (S2: 5'GGATGAGGAT**GCTTCTTCTCTGCGTCAAATTCTGGATTCTCAAAAAATGGAATGGCGTTC**

**TAACGCTGGTGGTTCT**TAACACATG*GAATTC*TGCAACAAC 3'; EcoRI site in italics) and used in a PCR fusion with F1 fragment using oligonucleotides containing respectively AflII and EcoRI sites (S3: 5' AGGCTGAACAT*CTTAAG*ACAGC 3', AS3: 5'TTGCA*GAATTC*CCGTTAAGAACC3'). The final PCR product was digested with AflII and EcoRI and was swapped for the corresponding fragment in pCMVΔR8.74. For pCMVΔR8.74-MA-BAD, a BstBI unique site was added by PCR to the 3' end of the MA at position 383 of the GAG coding sequence in the pCMVΔR8.74. A BstBI-BAD linker was made by annealing S4 (5'-PO4-*CGAA***GCTTCTTCTCTGCGTCAAATTCTGGATTCTCAAAAAATGGAATGGCGTTCTAACGCTGGTGGTTCT***TT*-3', BAD inderlined) and AS5 (5'-PO4-*GCTT***AGAACCACCAGCGTTAGAACGCCATTCCATTTTTTGAGAATCCAGAATTTGACGCAGAGAAGAAGC***AA*) which was ligated with the BstBI digested pCMVΔR8.74. The HA tag was introduced at the 3'-end of the pol gene of pCMVΔR8.74 by PCR using primers S1 and AS4 (5'GCA*GAATTC*CATGTGTTA**AGCGTAATCTGGAACATCGTATGGGTACAT**ATCCTCATCCTGTCTACT 3', HA tag underlined). The PCR product was digested with AflII and EcoRI and was swapped for the corresponding fragment in the pCMVΔR8.74. The Q168A mutation was introduced in pCMVΔR8.74-IN-BAD by PCR-directed mutagenesis, using the Quick change II site directed mutagenesis kit (Stratagene) and an oligonucleotide which contained GCG in place of the CAG codon in position 501 of the IN ORF (5' GGACAGGTAAGAGAT**GCG**GCTGAACATCTTAAGAC 3'). The HIV-1-derived self-inactivating pRRL-H1shRNA^LEDGF/p75^-PGK-eGFP-WPRE and pRRL-H1shRNA^ctl^-PGK-eGFP-WPRE transfer plasmids were constructed from a previously described system [44]. Sense siRNA sequences targeting LEDGF/p75 and control sequence were respectively AAAGACAGCATGAGGAAGCGA [20], TGTTTTAAGGGCCCCCCGT [44].

#### Cell culture

HEK 293T, HEK 293 and HCT116 cells were cultured in Dulbecco's modified eagle media (DMEM) supplemented with 10% foetal calf serum, 1% L-glutamine, 100 U/ml penicillin, and 100 μg/ml streptomycin (Gibco BRL) at 37°C, 5% CO_2_.

### Vector production and titrations

#### Production

VSV-G pseudotyped lentiviral vector encoding GFP were prepared by transient transfection into 293T cells [45]. For tagged vectors, gag-pol expression constructs with tagged (IN-BAD or IN-HA) IN sequence or tagged (MA-BAD) MA sequence were used. Briefly, cells were seeded into 15 cm dishes at 10^6 ^cells per dish and transfected 72 h later. A total of 60 μg of plasmid DNA was used for the transfection of one dish: 14.6 μg of the gag-pol construct, 7.9 μg of the envelope plasmid pMD.G, 22.5 μg of the transfer vector plasmid (pRRL-sin-PPT-hPGK-GFP-WPRE or pRRL-H1shRNA^LEDGF/p75^-PGK-eGFP-WPRE or pRRL-H1shRNA^ctl^-PGK-eGFP-WPRE). For biotinylation, 15 μg of the pcDNAbirA construct was included in IN-WT, IN-BAD or MA-BAD lentivector preparations. Vectors supernatants were collected every 24 h for 96 h and concentrated by ultracentrifugation (20.000 rpm, 2 h), aliquoted, and stored at -80°C until used.

#### Titrations

Titers of vector particles were obtained by measuring the number of transducing units (TU/ml) in FACS analysis after limiting dilution in HCT116 cells or the amount of p24 antigen released from the producing cells (not shown). TU/ml were calculated as the number of cells infected × percentage of GFP^+ ^cells/100 × dilution of vector. The p24 antigen concentration was determined by p24 core profile ELISA to estimate the titer of PP (physical particles) based on the assumption that 1fg of p24 represent 12pp [46].

### Vector transduction and cells extracts

All transductions were done with vectors that have equivalent TU/PP ratio. For proteins-BAD capture or immunoprecipitations, fifteen million HEK 293 cells were transduced (MOI 50) with IN-BAD or INQ168A-BAD or MA-BAD or IN-HA vectors or mock-transduced. When necessary, azidothymidine (AZT) was added 24 h before transduction at the final concentration of 100 μM. To remove vector excess, cells were washed two times with Phosphate Buffer Saline (PBS) 2 hours post-infection. Six hours later, cells were washed three times with K buffer (150 mM KCL, 20 mM HEPES [pH 7.6], 5 mM MgCl_2_, 0.5% [vol/vol] Triton X-100, 1 mM dithiothreitol supplemented with proteases and phosphatases inhibitors cocktail (Roche)) [6] without Triton X100 and cells extracts were prepared in 1 ml of K buffer.

For shRNA experiments, 10^6 ^HEK 293 cells were transduced at different MOI (10, 20, 30) in thenpresence of polybrene (4 μg/ml; Sigma Aldrich). After 3 rounds of transduction over a period of 48 h, cells were cultured for 3 weeks and enriched by sorting GFP^+ ^populations using flow cytometry. For the analysis of LEGDF/p75 protein expression, cells protein extracts were prepared from 10^7 ^cells that were lysed for 30 mn in K buffer. For Q-PCR, DNA samples were prepared with the Wizard Genomic DNA Extraction Kit (Promega).

### Biotinylation analysis

To analyse the IN biotinylation status, IN-BAD and IN-WT vector preparations were either directly loaded onto an SDS PAGE or lysed 30 mn in K buffer and incubated 2 hours with 20 μl of paramagnetic streptavidin-coated beads before material elution and loading (10^7 ^particles per lane). IN-BAD and IN-WT were revealed on Western blots probed with an anti-IN antibody (8G4, NIH AIDS Research and Reference Reagent Program) or an anti-biotin antibody (Tebu-bio).

For immunoprecipitations, 2.5 μg of LEDGF/p75 (Serotec) or p24 or MA (Tebu-bio) or HA (Roche) antibodies were incubated 2 hours with 20 μl of Protein A-coated beads in 100 μl of K buffer and washed three times to remove antibodies excess. 500 μl of cell lysates were incubated overnight with 20 μl of Protein A-coated beads pre-bound to the antibodies or with 20 μl of streptavidin-coated Dynabeads (Invitrogen) for BAD capture and the eluted material was analysed by Western blotting using the appropriate antibody.

### Q-PCR and PCR

#### Q-PCR

The kinetics of viral DNA synthesis and integration of IN-BAD or IN-WT vectors were determined by Q-PCR following transduction (30 ng of p24^gag ^antigen per 10^6 ^HEK 293 cells, MOI 10) as described previously [17]. The number of vector copies per cell of the pRRL-H1shRNA^LEDGF/p75^-PGK-eGFP-WPRE or the pRRL-H1shRNA^ctl^-PGK-eGFP-WPRE was determined by Q-PCR, amplifying from the genomic DNA the Woodchuck post-trancriptional regulatory element (WPRE) sequences of the lentiviral vector in comparison with the human albumin gene as previously described [44].

#### PCR

1/10 of beads of the streptavidin pull downs or the LEDGF/p75 co-immunoprecipitation were diluted in 10 μl of Tris/EDTA buffer and subjected to a PCR using the MH531 and MH532 oligonucleotides [17] to amplify total HIV-1 DNA. The HIV-1-derived self-inactivating pRRLsin-hPGK-eGFP-WPRE transfer plasmid was used as a positive control (not shown).

## Competing interests

The authors declare that they have no competing interests.

## Authors' contributions

SBZ has been involved in the supervising of the study, has trained and supervised JP and SB, designed experiments, conducted experiments with SB and JP, interpreted the data, and drafted the paper. SB has provided a substantial technical assistance. JP has carried out the shRNA experiments. AZB has designed and performed BAD constructions. OD has conceived of and supervised the study, and was involved in drafting the manuscript and revising it critically for intellectual content. All authors read and approved the final manuscript.
